# Competing Endogenous RNA Network Analysis Reveals Pivotal ceRNAs in Adrenocortical Carcinoma

**DOI:** 10.3389/fendo.2019.00301

**Published:** 2019-05-15

**Authors:** Weiwei Liang, Fangfang Sun

**Affiliations:** ^1^Department of Endocrinology, The Second Affiliated Hospital, Zhejiang University School of Medicine, Hangzhou, China; ^2^Cancer Institute (Key Laboratory of Cancer Prevention and Intervention, China National Ministry of Education; Key Laboratory of Molecular Biology in Medical Sciences), The Second Affiliated Hospital, Zhejiang University School of Medicine, Hangzhou, China

**Keywords:** adrenocortical carcinoma, non-coding RNA, ceRNA network analysis, lncRNA, ceRNA

## Abstract

**Objective:** To construct ceRNA network and identify pivotal competing endogenous RNAs (ceRNAs) in adrenocortical carcinoma (ACC) using ceRNA network analysis.

**Methods:** The RNA sequencing expression data of 77 ACCs in TCGA were obtained from GEPIA. Cancer specific ceRNAs, cancer specific microRNAs (miRNAs), and cancer specific messenger RNAs (mRNAs) were identified. The interaction of cancer specific miRNAs with cancer specific ceRNAs and cancer specific mRNAs were predicted. CeRNA network was constructed and visualized by Cytoscape 3.7.0 software. The genes in ceRNA network regulated GO terms and regulated pathways were performed by function analysis. Survival analysis of pivotal ceRNAs was performed for the pivotal lncRNAs.

**Result:** Twenty-eight cancer specific ceRNAs, 149 cancer specific miRNAs, and 104 mRNAs were identified. CeRNA network was constructed including 10 ceRNAs, 35 miRNAs, and 34 mRNAs. The genes in ceRNA network regulated GO terms and were classified into three groups: cellular component (CC), molecular function (MF), and biological process (BP). The genes in ceRNA network regulated the following pathways: leukocyte transendothelial migration, and proteoglycans in cancer. Survival analysis showed that CTB-63M22.1 and RP1-241P17.4 were significantly associated with ACC patient disease free survival and overall survival.

**Conclusion:** This study has constructed ceRNA networks in ACC. The study provides a set of pivotal ceRNAs for future investigation into the molecular mechanisms.

## Introduction

Cancers are often associated with aberrant transcriptomes. Recent analyses of the human transcriptome have revealed that 2% of the genome encode protein-coding transcripts even though over three-quarters of the genome are transcribed (1, 2). As genes do not function in isolation, they can be grouped into “networks” based on their interactions. A novel hypothesis, known as the ceRNA hypothesis presented by Salmena et al. (3), has emerged in recent years. CeRNAs, acting as miRNA sponges, can bind miRNAs competitively to indirectly regulate mRNAs using shared microRNA response elements. In theory, ceRNAs refer to all transcripts that may become the targets of miRNA such as long non-coding RNA (lncRNA), pseudogene RNA, and circular RNA. The function of miRNAs is relatively well-understood. MiRNAs can interact with target mRNAs to regulate the expression of the gene (4). Studies have shown that miRNAs are involved in the initiation and progression of cancers (4). While studies on lncRNAs and transcribed pseudogenes are still in their infancy, some studies showed that lncRNAs and pseudogenes played key regulatory roles in cancer biology (5). Recently, investigations about the ceRNA networks provide a better understanding of tumorigenesis (3, 6). This hypothesis laid the foundations for an important discovery regarding cross talk between both coding and non-coding RNAs through miRNAs.

Though an increasing number of miRNAs (7, 8) and lncRNAs (9) have been discovered to play a role in endocrine cancer. Our knowledge about the ceRNA network in endocrine cancer is still limited. The regulation landscape of ceRNA crosstalk in cancers is still largely unknown.

In this study, we focused on adrenocortical carcinoma (ACC). ACC is a rare endocrine malignancy, often with an unfavorable prognosis (10, 11); 5-years overall survival is below 40% (11). According to a report based on the Surveillance, Epidemiology, and End Results (SEER) database, the incidence of ACC is ~0.72 per million cases per year leading to 0.2% of all cancer deaths in the United States (12). The recent identification of the genomic alterations had provided some molecular mechanisms (13), but ceRNA crosstalk in ACC is still unknown.

In this study, we have taken full advantage of the rich data from the TCGA consortium. The aberrant expression profiles of RNA in ACC were filtered. The aberrant ceRNA network was constructed. This paper represented a significant leap forward in understanding the biological functions of ceRNAs in endocrine cancers.

## Materials and Methods

### Data Sources

The RNA sequencing expression data of 77 ACC patients in TCGA were obtained from GEPIA, a web server for cancer, normal gene expression profiling, and interactive analyses (http://gepia.cancer-pku.cn/index.html) (14). The dataset was strict to ACC, with [log_2_FC] > 3 and *p*-value < 0.01. ANOVA was used as differential methods. Both over-expressed and under-expressed expression data were downloaded.

### Construction of ceRNA Network of ACC

The RNA expression data downloaded from GEPIA were annotated by R package RefSeq mRNA and RefSeq ncRNA. We then used R package transcript biotype to double check.

First, we identified cancer specific ceRNA, cancer specific miRNA, and cancer specific mRNA. The traditional view was that lncRNA functioned as a ceRNA. Recent articles suggested that pseudogenes also have ceRNA function (15). Therefore, in this paper lncRNA and pseudogenes were both identified as potential cancer specific ceRNAs. Cancer specific miRNAs were retained from Oncomir (http://www.oncomir.org) (16). A list of miRNAs closely associated with patient survival or tumor development were retrieved with *p* < 0.05. mRNAs with absolute [log_2_FC] > 3 and *p*-value < 0.01 retained from GEPIA were identified as cancer specific mRNAs.

The ceRNA network was then constructed through the following steps: (1) Prediction of cancer specific ceRNA-cancer specific miRNA interaction: miRcode (http://www.mircode.org) and DIANA-LncBase v2 (www.microrna.gr/LncBase) (17) was used to predict cancer specific ncRNA-cancer specific miRNA interaction. (2) Cancer specific miRNA-cancer specific mRNA interaction: miRtarBase (http://mirtarbase.mbc.nctu.edu.tw/php/index.php) (18) was used to retrieve cancer specific miRNA-cancer specific miRNA interaction. (3) CeRNA network construction and visualization; Cytoscape 3.7.0 software (19) was utilized to construct and visualize ceRNA network.

### Functional Analysis

The biological function and pathway of the genes involved in the ceRNA network would demonstrate instructive information. Thus, we used Gene ontology analysis (GO) to identify characteristic biological attributes. Kyoto Encyclopedia of Genes and Genomes pathway (KEGG) enrichment analysis was performed to identify functional attributes. *p* < 0.05 was set as the cut-off criterion.

### Survival Analysis of Pivotal lncRNAs

The ceRNAs in ACC ceRNA network were identified as pivotal ceRNAs. CeRNAs' correlations with patient survival were featured in GEPIA. Available TCGA patient survival data were used for Kaplan–Meier survival analysis and to generate overall and disease-free survival plots. Group-cutoff was set as the median, the hazards ratio was calculated based on Cox PH Model and the 95% confidence interval was added as a dotted line. *p* < 0.05 was set as the cut-off criterion.

## Results

### Identification of Cancer Specific ceRNAs, miRNAs, and mRNAs

Seventy-seven ACC patient data in TCGA were analyzed. Twenty-eight cancer specific ceRNAs (10 down regulated in ACC and 18 up regulated in ACC, Supplementary Table 1), 149 cancer specific miRNAs and 104 cancer specific mRNAs were identified (Supplementary Tables 2,3).

### CeRNA Network Construction

**Figure 1:**
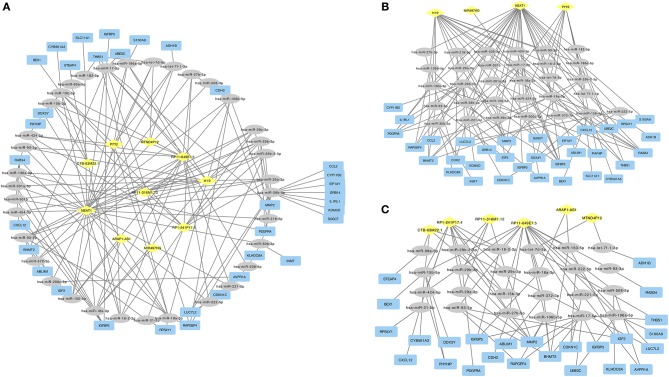
**(A)** ceRNA network in ACC. Diamond in yellow represented cancer specific ceRNA. Ellipse in gray represented cancer specific mRNA. Rectangle in blue represented cancer specific mRNA. **(B)** Down-regulated ceRNAs regulated ceRNA network. **(C)** Up-regulated ceRNAs regulated ceRNA network.

To construct the ceRNA network, we next predicted the interaction of cancer specific miRNAs with cancer specific ceRNAs and cancer specific mRNAs. We overlapped the predicted targets of cancer specific ceRNAs with cancer specific miRNAs and overlapped the predicted targets of cancer specific miRNAs with cancer specific mRNAs. Finally, the ceRNA regulatory network was constructed (Figure 1A), including 10 ceRNAs, 35 miRNAs, and 34 mRNAs. In the down-regulated ceRNA regulatory network, there were 4 ceRNAs, 31 miRNAs, and 33 mRNAs involved (Figure 1B). And in the up-regulated ceRNA regulatory network, there were six ceRNAs, 24 miRNAs, and 25 mRNAs involved (Figure 1C).

### Gene Ontology and Pathway Enrichment Analyses

**Figure 2:**
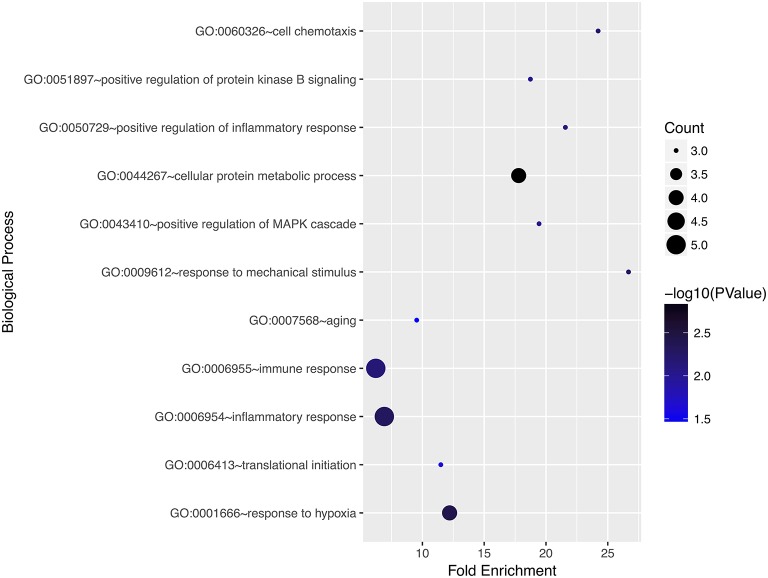
Gene ontology analysis for biological process of genes involved in the ceRNA network in ACC.

**Figure 3:**
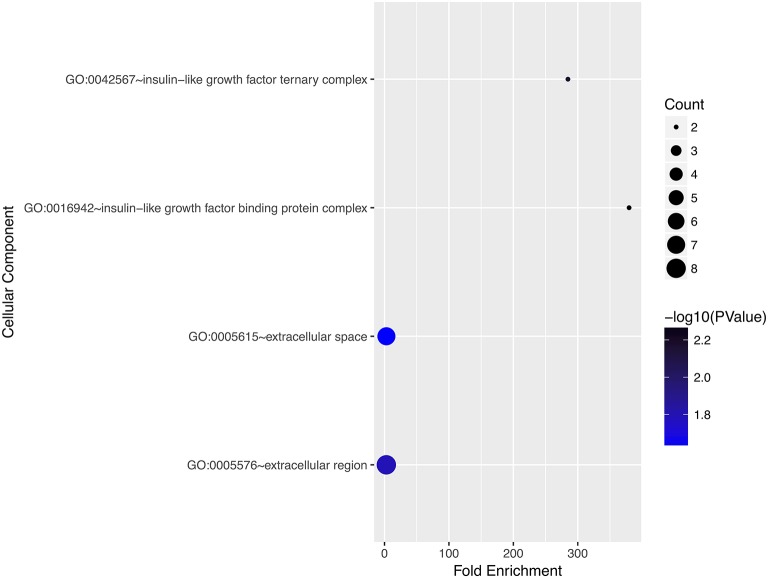
Gene ontology analysis for cellular component of genes involved in the ceRNA network in ACC.

**Figure 4:**
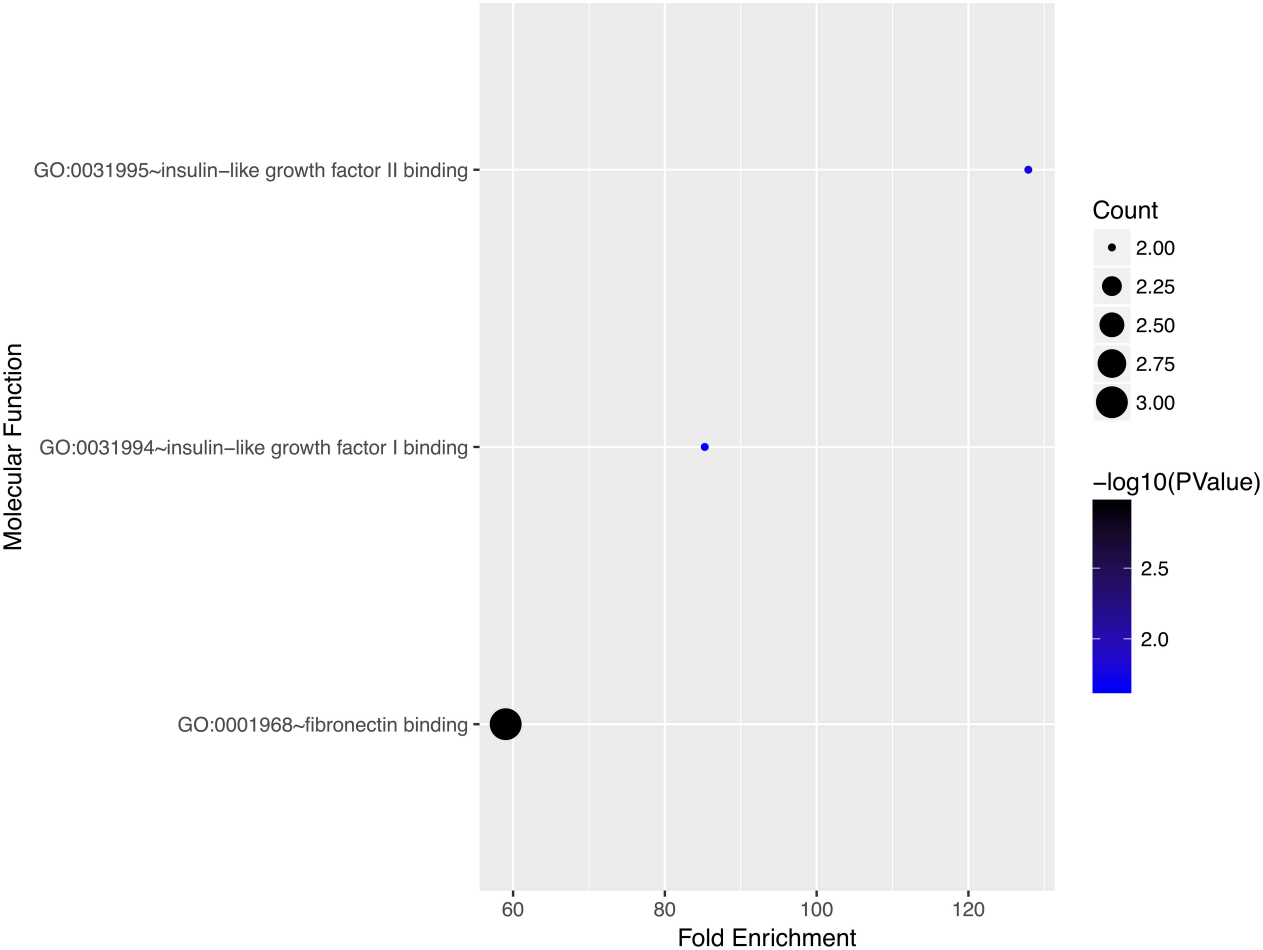
Gene ontology analysis for molecular function of genes involved in the ceRNA network in ACC.

We performed gene ontology analysis of the genes in the ceRNA network (Figures 2–4, Tables 1–3). The result showed that related to CC, the genes in the ceRNA network were mainly enriched in extracellular region. Related to MF, the genes in the ceRNA network were mainly enriched in fibronectin binding. Related to BP, the genes in the ceRNA network were mainly enriched in inflammatory response and immune response.

**Table 1:** Gene ontology analysis for biological process of genes involved in the ceRNA regulation network in ACC.

**Term**	**Genes**	***P*-value**	**Fold enrichment**	**FDR**
GO:0009612~response to mechanical stimulus	*CCL2, THBS1, CXCL12*	5.29E-03	26.68	7.28
GO:0060326~cell chemotaxis	*CCL2, PDGFRA, CXCL12*	6.38E-03	24.22	8.72
GO:0050729~positive regulation of inflammatory response	*CCL2, IL1RL1, S100A9*	7.99E-03	21.57	10.80
GO:0043410~positive regulation of MAPK cascade	*IGF2, CDH2, IGFBP3*	9.76E-03	19.44	13.05
GO:0051897~positive regulation of protein kinase B signaling	*IGF2, THBS1, IGFBP5*	1.05E-02	18.74	13.93
GO:0006413~translational initiation	*EIF1AY, DDX3Y, RPS4Y1*	2.63E-02	11.49	31.64
GO:0007568~aging	*CDKN1C, CCL2, IGFBP5*	3.70E-02	9.54	41.61
GO:0044267~cellular protein metabolic process	*IGF2, IGFBP3, MMP2, IGFBP5*	1.32E-03	17.79	1.86
GO:0001666~response to hypoxia	*CCL2, THBS1, CXCL12, MMP2*	3.85E-03	12.20	5.34
GO:0006954~inflammatory response	*SLC11A1, CCL2, S100A9, THBS1, CXCL12*	4.96E-03	6.92	6.85
GO:0006955~immune response	*SLC11A1, CCL2, IL1RL1, THBS1, CXCL12*	7.17E-03	6.23	9.75

**Table 2:** Gene ontology analysis for cellular component of genes involved in the ceRNA regulation network in ACC.

**Term**	**Genes**	***P*-value**	**Fold enrichment**	**FDR**
GO:0016942~insulin-like growth factor binding protein complex	*IGFBP3, IGFBP5*	5.09E-03	379.67	5.29
GO:0042567~insulin-like growth factor ternary complex	*IGFBP3, IGFBP5*	6.79E-03	284.75	6.98
GO:0005615~extracellular space	*CCL2, S100A9, IGF2, THBS1, CXCL12, IGFBP3, MMP2*	2.40E-02	2.96	22.77
GO:0005576~extracellular region	*CCL2, S100A9, IGF2, THBS1, CXCL12, IGFBP3, MMP2, IGFBP5*	1.65E-02	2.83	16.20

**Table 3:** Gene ontology analysis for molecular function of genes involved in the ceRNA regulation network in ACC.

**Term**	**Genes**	***P*-value**	**Fold enrichment**	**FDR**
GO:0031995~insulin-like growth factor II binding	*IGFBP3, IGFBP5*	1.51E-02	127.89	16.02
GO:0031994~insulin-like growth factor I binding	*IGFBP3, IGFBP5*	2.25E-02	85.26	23.04
GO:0001968~fibronectin binding	*THBS1, IGFBP3, IGFBP5*	1.10E-03	59.02	1.26

We then performed pathway enrichment analysis of the genes in the ceRNA network and identified the ceRNAs regulated pathway. Functional analysis showed that the ceRNAs regulated pathways included leukocyte transendothelial migration, and proteoglycans in cancer.

### Survival Analysis of Pivotal lncRNAs

**Figure 5:**
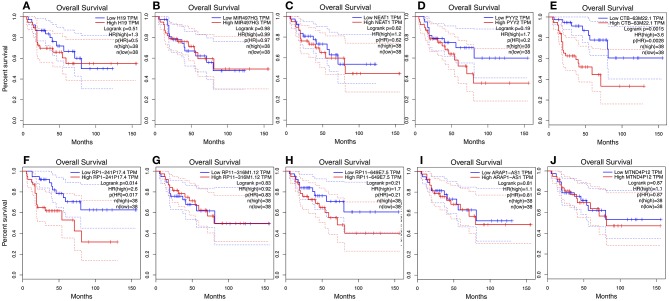
**(A–J)**: Overall survival plot of 10 pivotal ceRNAs using 77 ACC patient data from TCGA database.

**Figure 6:**
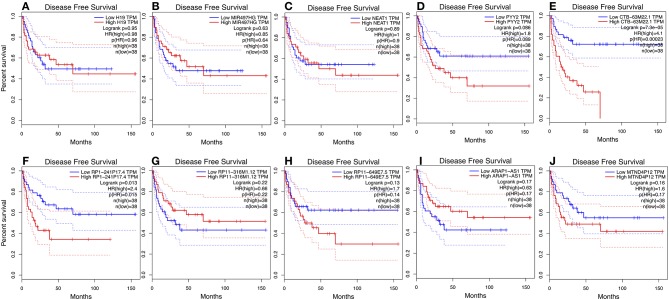
**(A–J)**: Disease free survival plot of 10 pivotal ceRNAs using 77 ACC patient data from TCGA database.

We used GEPIA to perform survival analysis of pivotal ceRNAs (Figures 5, 6). *p* < 0.05 was set as the cut-off criterion. The results showed that CTB-63M22.1 and RP1-241P17.4 were significantly associated with ACC patient disease free survival and overall survival.

## Discussion

Salmena et al. (3) firstly proposed a hypothesis on how mRNAs and lncRNAs “talk” to each other using miRNA as letters of a new language. The last few years have nominated ceRNA hypothesis as a novel layer of gene regulation. This ceRNA regulatory network greatly expands the functional genetic information and will aid in deciphering the underlying pathogenesis of human polygenic disease at the post-transcriptional level. Previously, Xiao et al. (20) have analyzed the gene expression data of ACC in The Cancer Genome Atlas (TCGA) and identified the potential prognostic mRNA biomarkers. To our knowledge, this is the first study to investigate the ceRNA network in ACC.

In this study, we extracted RNA expression profile from cases of ACC from TCGA. Twenty-eight cancer specific ceRNAs, 149 cancer specific miRNAs, and 104 mRNAs were identified. A ceRNA network was then constructed, consisting of 10 ceRNAs, 35 miRNAs, and 34 miRNAs. The Kyoto Encyclopedia of Genes and Genomes (KEGG), and the Gene Ontology (GO) databases were used to identify biological and functional attributes of the genes in the ceRNA network. The ceRNA network-regulated GO terms were classified into three groups. The ceRNA network was mainly enriched in the following pathways: leukocyte transendothelial migration and proteoglycans in cancer. Survival analysis showed two potential prognostic associated ceRNAs: CTB-63M22.1 and RP1-241P17.4.

Of the genes that were involved in the ceRNA network, GO functional enrichment analysis showed that these genes were mainly enriched in the extracellular region, fibronectin binding, inflammatory response, and immune response. Perge's (21) previous study showed that miRNAs in exosome (an extracellular substance) might have potential diagnostic value in ACC. MiR-31 in serum exosomes possibly correlated with an aggressive profile in endocrine cancer metastasis (8). Interestingly, miR-31 was a miRNA identified in our ceRNA network. Griggs et al. (22) reported that fibronectin, a protein in the extracellular matrix, served as a growth factor delivery system in epithelial-mesenchymal transition of cancers. Host immune response status played an important role in the clinical outcome of patients with cancer. Systemic inflammatory response was related with cancer progression (23, 24).

Of the genes that were involved in ceRNA network, KEGG analysis showed that these genes were mainly enriched in leukocyte transendothelial migration and proteoglycans in cancer. Leukocyte transendothelial migration is generally activated in cancer progression (25). Proteoglycans (PGs), comprising structurally diverse constituents of the extracellular matrix and cell surfaces, have emerged as novel biomarkers and molecular players both within tumor cells and within their microenvironment. Recent studies have demonstrated that the expression of proteoglycans is regulated by miRNAs (26, 27). The ceRNA network identified in our study provided useful clues of further study.

By analyzing TCGA data, 35 cancer specific miRNA were identified in the ceRNA network. Several miRNAs in our list have been investigated in ACC: Doghman et al. (28) identified that miR-99a was differentially expressed in childhood adrenocortical tumors. Mir-99a might involve in tumor cell growth via regulating mTOR signaling. Bimpak et al. (29) found miR-200 was differentially expressed in massive macronodular adrenocortical disease and miR-200b directly targeted matrin 3 expression in an adrenocortical cancer cell line. The understanding of other miRNAs identified in this study is limited, therefore, further molecular studies are require. These ACC-specific miRNAs might become potential biomarkers with specificity in the diagnosis and progression of ACC in future.

In our study, we identified 10 pivotal ceRNAs. Liu's study showed that H19 expression is maintained at low levels in hormonally active adrenocortical carcinomas, which is consistent with our study (30). There is some evidence indicating that the ceRNAs identified in our study are associated with other cancer types. Xiong et al. (31) reported that NEAT1, a ceRNA, accelerated lung adenocarcinoma deterioration. Teng et al.'s (32) recent study showed that lncRNA ARAP1-AS1, which functioned as a ceRNA, promoted the progression of bladder cancer by regulating the miR-4735-3p/NOTCH2 axis. Studies of other ceRNAs are limited, but they provide a good direction for future research. Of the 10 pivotal ceRNAs, CTB-63M22.1, and RP1-241P17.4 were associated with tumor prognosis. There are currently no studies on these two ceRNAs, so they are worthy of future research.

Although the findings of our study have important clinical implications, the limitations must also be noted. In addition to lncRNA and pseudogenes, cirRNA and tRNA may also function as ceRN, and their role in ceRNA network needs further research. Further studies with much larger sample sizes and longer follow-up are required to verify our results. The biological roles of ceRNAs in ACC need to be further investigated. In the future, molecular biology methods including qPCR, luciferase reporter systems and co-immunoprecipitation assays are helpful to validate our findings, thus unraveling the molecular mechanisms of ceRNA networks. CeRNA activity is associated with a multitude of parameters such as the number of miRNA binding types residing on coding and non-coding transcripts, and these parameters need further investigation.

In conclusion, this study has constructed ceRNA network in ACC. The study provides a set of pivotal ceRNAs for future investigation into the molecular mechanisms. This study provided novel insights to explore the underlying mechanism of endocrine cancers.

## Author Contributions

WL drew the figures and wrote the paper. FS analyzed the data.

### Conflict of Interest Statement

The authors declare that the research was conducted in the absence of any commercial or financial relationships that could be construed as a potential conflict of interest.
