# Supplementary material for: Competing Endogenous RNA Network Analysis Reveals Pivotal ceRNAs in Adrenocortical Carcinoma
Source: Front Endocrinol (Lausanne). 2019 May 15;10:301. doi: 10.3389/fendo.2019.00301 (PMC6529643; doi:10.3389/fendo.2019.00301)
Supplement: Supplementary file 1 [file Table_1.docx]

| ID | Transcript_ID | log2FC | Pvalue |
| --- | --- | --- | --- |
| TMSB4XP6 | ENSG00000230043 | -4.855 | 1.28E-12 |
| TXLNGY | ENSG00000131002 | -4.199 | 1.52E-07 |
| H19 | ENSG00000130600 | -3.721 | 7.79E-23 |
| PAX8-AS1 | ENSG00000189223 | -3.473 | 2.73E-17 |
| GSTA9P | ENSG00000243236 | -3.461 | 8.98E-16 |
| NEAT1 | ENSG00000245532 | -3.387 | 2.27E-41 |
| PYY2 | ENSG00000237575 | -3.32 | 2.13E-46 |
| MIR497HG | ENSG00000267532 | -3.206 | 1.84E-55 |
| RP11-802E16.3 | ENSG00000255031 | -3.099 | 1.19E-58 |
| CTB-79E8.3 | ENSG00000253683 | -3.065 | 1.61E-56 |
| RP11-649E7.5 | ENSG00000258377 | 3.025 | 6.53E-33 |
| CTC-518P12.6 | ENSG00000267484 | 3.095 | 2.12E-55 |
| AC005786.7 | ENSG00000267436 | 3.109 | 2.69E-16 |
| RP11-667K14.4 | ENSG00000262533 | 3.302 | 1.94E-46 |
| UBE2SP2 | ENSG00000224126 | 3.437 | 4.05E-66 |
| MTND4P12 | ENSG00000247627 | 3.616 | 3.35E-16 |
| RP11-498C9.3 | ENSG00000262413 | 3.718 | 7.06E-21 |
| RP11-256P1.1 | ENSG00000249971 | 3.741 | 1.29E-25 |
| RP5-890E16.4 | ENSG00000266341 | 3.751 | 3.57E-54 |
| ARAP1-AS1 | ENSG00000256007 | 3.779 | 2.23E-16 |
| HSPB1P1 | ENSG00000236060 | 3.922 | 1.81E-73 |
| LLNLR-284B4.1 | ENSG00000274177 | 4.075 | 1.41E-29 |
| CTC-425F1.4 | ENSG00000267458 | 4.127 | 2.94E-39 |
| CTB-63M22.1 | ENSG00000229119 | 4.906 | 5.63E-78 |
| RP11-316M1.12 | ENSG00000259357 | 5.347 | 2.14E-51 |
| AC132217.4 | ENSG00000240801 | 6.822 | 1.44E-40 |
| RP5-940J5.9 | ENSG00000269968 | 7.983 | 2.38E-46 |
| RP11-40C6.2 | ENSG00000219928 | 8.955 | 5.81E-117 |

**Sup. Table 1:** 28 cancer specific ceRNAs of ACC identified from GEPIA.
