# Supplementary material for: Competing Endogenous RNA Network Analysis Reveals Pivotal ceRNAs in Adrenocortical Carcinoma
Source: Front Endocrinol (Lausanne). 2019 May 15;10:301. doi: 10.3389/fendo.2019.00301 (PMC6529643; doi:10.3389/fendo.2019.00301)
Supplement: Supplementary file 2 [file Table_2.docx]

| MiRNA name |
| --- |
| hsa-let-7a-2-3p, hsa-let-7d-5p, hsa-let-7f-1-3p, hsa-let-7i-3p, hsa-miR-101-3p, hsa-miR-101-5p, hsa-miR-106b-3p, hsa-miR-106b-5p, hsa-miR-1246, hsa-miR-1251-5p, hsa-miR-1255a, hsa-miR-1258, hsa-miR-125a-5p, hsa-miR-125b-1-3p, hsa-miR-125b-2-3p, hsa-miR-1262, hsa-miR-1296-5p, hsa-miR-1298-5p, hsa-miR-1301-3p, hsa-miR-1304-5p, hsa-miR-1306-3p, hsa-miR-1307-3p, hsa-miR-130a-3p, hsa-miR-130b-3p, hsa-miR-130b-5p, hsa-miR-135b-5p, hsa-miR-141-3p, hsa-miR-141-5p, hsa-miR-150-5p, hsa-miR-15b-3p, hsa-miR-15b-5p, hsa-miR-16-2-3p, hsa-miR-17-5p, hsa-miR-181b-5p, hsa-miR-182-5p, hsa-miR-183-5p, hsa-miR-18a-3p, hsa-miR-18a-5p, hsa-miR-1914-5p, hsa-miR-196a-5p, hsa-miR-196b-5p, hsa-miR-197-3p, hsa-miR-1976, hsa-miR-199b-5p, hsa-miR-19a-3p, hsa-miR-19b-1-5p, hsa-miR-200c-3p, hsa-miR-20a-3p, hsa-miR-212-3p, hsa-miR-214-3p, hsa-miR-218-5p, hsa-miR-221-5p, hsa-miR-222-5p, hsa-miR-25-3p, hsa-miR-26b-3p, hsa-miR-26b-5p, hsa-miR-27a-5p, hsa-miR-27b-5p, hsa-miR-29a-3p, hsa-miR-29b-2-5p, hsa-miR-29b-3p, hsa-miR-29c-3p, hsa-miR-29c-5p, hsa-miR-301a-3p, hsa-miR-301b, hsa-miR-30b-3p, hsa-miR-3127-5p, hsa-miR-3136-5p, hsa-miR-31-3p, hsa-miR-3144-3p, hsa-miR-31-5p, hsa-miR-3170, hsa-miR-3176, hsa-miR-320b, hsa-miR-324-5p, hsa-miR-32-5p, hsa-miR-326, hsa-miR-329-3p, hsa-miR-330-3p, hsa-miR-330-5p, hsa-miR-331-5p, hsa-miR-33a-3p, hsa-miR-3605-5p, hsa-miR-361-5p, hsa-miR-362-5p, hsa-miR-3677-3p, hsa-miR-372-3p, hsa-miR-374b-3p, hsa-miR-376a-3p, hsa-miR-3917, hsa-miR-3923, hsa-miR-3928-3p, hsa-miR-3934-5p, hsa-miR-3940-3p, hsa-miR-424-3p, hsa-miR-424-5p, hsa-miR-425-5p, hsa-miR-454-3p, hsa-miR-454-5p, hsa-miR-466, hsa-miR-503-5p, hsa-miR-506-3p, hsa-miR-507, hsa-miR-508-3p, hsa-miR-508-5p, hsa-miR-509-3-5p, hsa-miR-509-3p, hsa-miR-509-5p, hsa-miR-510-5p, hsa-miR-513a-3p, hsa-miR-513a-5p, hsa-miR-513b-5p, hsa-miR-513c-5p, hsa-miR-514a-3p, hsa-miR-514b-3p, hsa-miR-514b-5p, hsa-miR-550a-3p, hsa-miR-550a-5p, hsa-miR-573, hsa-miR-574-3p, hsa-miR-576-5p, hsa-miR-581, hsa-miR-585-3p, hsa-miR-590-5p, hsa-miR-615-3p, hsa-miR-618, hsa-miR-651-5p, hsa-miR-653-5p, hsa-miR-664a-3p, hsa-miR-664a-5p, hsa-miR-676-3p, hsa-miR-7-1-3p, hsa-miR-744-3p, hsa-miR-769-3p, hsa-miR-874-3p, hsa-miR-877-5p, hsa-miR-887-3p, hsa-miR-92b-3p, hsa-miR-93-3p, hsa-miR-93-5p, hsa-miR-937-3p, hsa-miR-9-3p, hsa-miR-940, hsa-miR-942-5p, hsa-miR-944, hsa-miR-9-5p, hsa-miR-96-5p, hsa-miR-99a-3p, hsa-miR-99a-5p |

**Sup. Table 2:** List of 149 cancer specific miRNAs of ACC.
