# Supplementary material for: Competing Endogenous RNA Network Analysis Reveals Pivotal ceRNAs in Adrenocortical Carcinoma
Source: Front Endocrinol (Lausanne). 2019 May 15;10:301. doi: 10.3389/fendo.2019.00301 (PMC6529643; doi:10.3389/fendo.2019.00301)
Supplement: Supplementary file 3 [file Table_3.docx]

| ID | Transcript_ID | log2FC | Pvalue |
| --- | --- | --- | --- |
| RPS4Y1 | ENSG00000129824 | -7.664 | 0.000269 |
| AADAC | ENSG00000114771 | -6.626 | 2.67E-60 |
| PTGDS | ENSG00000107317 | -6.285 | 2.00E-41 |
| HSD3B2 | ENSG00000203859 | -5.328 | 6.98E-31 |
| KDM5D | ENSG00000012817 | -5.139 | 5.81E-09 |
| ADH1B | ENSG00000196616 | -5.084 | 8.87E-48 |
| DDX3Y | ENSG00000067048 | -4.86 | 3.89E-07 |
| CYP11B1 | ENSG00000160882 | -4.825 | 5.32E-27 |
| DCN | ENSG00000011465 | -4.816 | 8.01E-37 |
| ADGRV1 | ENSG00000164199 | -4.648 | 3.02E-39 |
| REG3G | ENSG00000143954 | -4.523 | 7.05E-25 |
| SAA1 | ENSG00000173432 | -4.501 | 5.15E-33 |
| DAPL1 | ENSG00000163331 | -4.47 | 2.11E-32 |
| TMBIM4 | ENSG00000282031 | -4.397 | 3.14E-42 |
| EIF1AY | ENSG00000198692 | -4.289 | 2.75E-05 |
| KCNJ5 | ENSG00000120457 | -4.269 | 2.80E-30 |
| KCNK2 | ENSG00000082482 | -4.224 | 1.15E-43 |
| AL442127.1 | ENSG00000278371 | -4.208 | 9.38E-23 |
| FBLN1 | ENSG00000077942 | -4.166 | 9.56E-39 |
| ABLIM1 | ENSG00000099204 | -4.102 | 1.55E-38 |
| LUM | ENSG00000139329 | -4.085 | 6.52E-33 |
| CDH2 | ENSG00000170558 | -4.056 | 8.82E-34 |
| INMT | ENSG00000241644 | -4.054 | 4.90E-61 |
| SLC40A1 | ENSG00000138449 | -3.987 | 2.07E-19 |
| IL1RL1 | ENSG00000115602 | -3.921 | 2.96E-51 |
| SLC37A2 | ENSG00000134955 | -3.921 | 1.03E-50 |
| S100A9 | ENSG00000163220 | -3.895 | 1.41E-33 |
| CYP11B2 | ENSG00000179142 | -3.851 | 1.74E-20 |
| USP9Y | ENSG00000114374 | -3.835 | 9.65E-08 |
| AVPR1A | ENSG00000166148 | -3.798 | 7.79E-40 |
| DUOX1 | ENSG00000137857 | -3.776 | 1.06E-65 |
| KCNQ1 | ENSG00000053918 | -3.74 | 1.53E-51 |
| SLC11A1 | ENSG00000018280 | -3.723 | 1.28E-59 |
| ANGPTL1 | ENSG00000116194 | -3.679 | 5.05E-31 |
| PHYHD1 | ENSG00000175287 | -3.674 | 3.09E-44 |
| SULT1E1 | ENSG00000109193 | -3.658 | 1.86E-55 |
| STEAP4 | ENSG00000127954 | -3.648 | 2.60E-48 |
| MARCO | ENSG00000019169 | -3.537 | 6.62E-29 |
| IGFBP6 | ENSG00000167779 | -3.528 | 1.29E-41 |
| PHYHIP | ENSG00000168490 | -3.483 | 1.48E-55 |
| NPM2 | ENSG00000158806 | -3.478 | 1.97E-45 |
| FAM65C | ENSG00000042062 | -3.473 | 2.45E-47 |
| ITIH4 | ENSG00000055955 | -3.459 | 3.12E-42 |
| RARRES1 | ENSG00000118849 | -3.45 | 1.78E-39 |
| CD163 | ENSG00000177575 | -3.429 | 1.49E-35 |
| CCL21 | ENSG00000137077 | -3.415 | 3.01E-19 |
| RAB34 | ENSG00000109113 | -3.412 | 2.40E-25 |
| PTH1R | ENSG00000160801 | -3.41 | 2.76E-52 |
| KLHDC8A | ENSG00000162873 | -3.403 | 8.81E-26 |
| S100A8 | ENSG00000143546 | -3.39 | 1.96E-31 |
| THBS1 | ENSG00000137801 | -3.385 | 4.43E-43 |
| LRRC38 | ENSG00000162494 | -3.382 | 3.99E-23 |
| LMOD1 | ENSG00000163431 | -3.365 | 3.23E-33 |
| IGFBP5 | ENSG00000115461 | -3.358 | 7.06E-46 |
| LUC7L2 | ENSG00000269955 | -3.343 | 2.23E-23 |
| RAPGEF4 | ENSG00000091428 | -3.328 | 1.53E-46 |
| COL4A3 | ENSG00000169031 | -3.317 | 1.36E-52 |
| ADAMTSL2 | ENSG00000197859 | -3.299 | 1.02E-64 |
| CYB561A3 | ENSG00000162144 | -3.266 | 3.75E-30 |
| CCL2 | ENSG00000108691 | -3.263 | 5.74E-36 |
| MT1A | ENSG00000205362 | -3.245 | 2.01E-19 |
| MFAP4 | ENSG00000166482 | -3.242 | 1.37E-20 |
| MRAP | ENSG00000170262 | -3.235 | 6.03E-26 |
| NOV | ENSG00000136999 | -3.235 | 2.79E-16 |
| AMT | ENSG00000145020 | -3.23 | 1.19E-56 |
| ZBED6CL | ENSG00000188707 | -3.219 | 1.73E-36 |
| GPX3 | ENSG00000211445 | -3.217 | 3.96E-34 |
| PRSS2 | ENSG00000275896 | -3.21 | 2.21E-34 |
| KLHL4 | ENSG00000102271 | -3.201 | 2.95E-25 |
| BHMT2 | ENSG00000132840 | -3.187 | 1.08E-38 |
| FCGR2C | ENSG00000244682 | -3.174 | 3.02E-45 |
| SLCO2A1 | ENSG00000174640 | -3.172 | 6.29E-59 |
| TRIM54 | ENSG00000138100 | -3.167 | 3.39E-36 |
| GRB14 | ENSG00000115290 | -3.158 | 1.13E-27 |
| CHRD | ENSG00000090539 | -3.153 | 5.91E-49 |
| RSPO3 | ENSG00000146374 | -3.151 | 3.19E-41 |
| F13A1 | ENSG00000124491 | -3.149 | 1.07E-25 |
| NPY1R | ENSG00000164128 | -3.144 | 2.85E-51 |
| SLCO2B1 | ENSG00000137491 | -3.082 | 2.82E-43 |
| SCNN1A | ENSG00000111319 | -3.07 | 2.52E-42 |
| PDGFRA | ENSG00000134853 | -3.068 | 2.41E-47 |
| PGA3 | ENSG00000229859 | -3.066 | 1.08E-37 |
| RP11-162P23.2 | ENSG00000257767 | -3.06 | 1.34E-19 |
| RALYL | ENSG00000184672 | -3.055 | 1.16E-31 |
| ALDH1A1 | ENSG00000165092 | -3.051 | 8.14E-18 |
| CDKN1C | ENSG00000129757 | -3.05 | 4.88E-22 |
| SPON2 | ENSG00000159674 | -3.048 | 9.44E-36 |
| SUGCT | ENSG00000175600 | -3.043 | 2.30E-41 |
| SPON1 | ENSG00000262655 | -3.042 | 1.13E-59 |
| MMP2 | ENSG00000087245 | -3.01 | 1.49E-40 |
| CXCL12 | ENSG00000107562 | -3.008 | 8.99E-34 |
| VSIG4 | ENSG00000155659 | -3.007 | 7.67E-26 |
| GIPC2 | ENSG00000137960 | -3.002 | 8.49E-31 |
| CES4A | ENSG00000172824 | -3.001 | 1.43E-57 |
| AC005943.6 | ENSG00000279009 | 3.032 | 7.30E-11 |
| ESM1 | ENSG00000164283 | 3.158 | 1.07E-49 |
| HSPB8 | ENSG00000152137 | 3.158 | 5.32E-18 |
| UBE2C | ENSG00000175063 | 3.219 | 2.41E-31 |
| BEX1 | ENSG00000133169 | 3.464 | 1.09E-10 |
| IGFBP3 | ENSG00000146674 | 3.522 | 1.71E-49 |
| BIRC7 | ENSG00000101197 | 3.678 | 4.85E-27 |
| MT3 | ENSG00000087250 | 3.744 | 8.65E-18 |
| NPTX2 | ENSG00000106236 | 4.168 | 6.94E-31 |
| IGF2 | ENSG00000167244 | 6.824 | 1.08E-41 |

**Sup. Table 3:** 104 cancer specific mRNAs of ACC identified from GEPIA.
